# Conventional radiography in juvenile idiopathic arthritis: Joint recommendations from the French societies for rheumatology, radiology and paediatric rheumatology

**DOI:** 10.1007/s00330-018-5304-7

**Published:** 2018-03-26

**Authors:** Pauline Marteau, Catherine Adamsbaum, Linda Rossi-Semerano, Michel De Bandt, Irène Lemelle, Chantal Deslandre, Tu Anh Tran, Anne Lohse, Elisabeth Solau-Gervais, Christelle Sordet, Pascal Pillet, Brigitte Bader-Meunier, Julien Wipff, Cécile Gaujoux-Viala, Sylvain Breton, Valérie Devauchelle-Pensec

**Affiliations:** 1 Service de rhumatologie, CHU de Brest, Brest, France; 2 Paediatric Radiology, Hôpital Bicêtre, Paris, France; 3 Paris Sud University Hôpital Bicêtre, Le Kremlin Bicêtre APHP, Paris, France; 4 Paediatric Rheumatology, Reference Centre for Autoinflammatory Diseases, Hôpital Bicêtre, AP-HP, Le Kremlin Bicêtre, France; 5 Rheumatology, Martinique University Hospital, P Zobda-Quitman Hospital, Route de Chateauboeuf, 97200 Martinique FWI, France; 6 Paediatric Onco-Haematology, CHRU Nancy, 5 Allée du Morvan, 54500 Vandoeuvre les Nancy, France; 7 Rheumatology A, Cochin Hospital, APHP, Paris, France; 8 Université René Descartes Paris 5, Paris, France; 9 Paediatrics, University Hospital, Nîmes, France; 10 INSERM U 1183, Montpellier University, Montpellier, France; 11 Rheumatology, Nord Franche Comte Hospital, CHBM 14 rue de Mulhouse, 9000 Belfort, France; 12 Rheumatology, Poitiers University Hospital, Poitiers, France; 13 Rheumatology, Hautepierre Hospital, Strasbourg, France; 14 Paediatrics, Pellegrin-Enfants, place Amélie Raba Léon, 33076 Bordeaux cedex, France; 15 Paediatric Rheumatology, Hôpital Necker, Paris, France; 16 Rheumatology A, Cochin Hospital, Paris, France; 17 Rheumatology, Carémeau University Hospital, 30029 Nîmes cedex 9, France; 18 Paediatric Radiology, Necker-Enfants Malades Hospital, Assistance Publique Hôpitaux de Paris, 149 rue de Sèvres, 75743 Paris Cedex 15, France; 19 Lymphocytes B et Autoimmunité, Université de Bretagne Occidentale, LabEx IGO, UMR1227, Brest, France

**Keywords:** Juvenile idiopathic arthritis, Conventional radiography, Recommendations, Structural damage, Erosions

## Abstract

**Background:**

Juvenile idiopathic arthritis (JIA) can cause structural damage. However, data on conventional radiography (CR) in JIA are scant.

**Objective:**

To provide pragmatic guidelines on CR in each non-systemic JIA subtype.

**Methods:**

A multidisciplinary task force of 16 French experts (rheumatologists, paediatricians, radiologists and one patient representative) formulated research questions on CR assessments in each non-systemic JIA subtype. A systematic literature review was conducted to identify studies providing detailed information on structural joint damage. Recommendations, based on the evidence found, were evaluated using two Delphi rounds and a review by an independent committee.

**Results:**

74 original articles were included. The task force developed four principles and 31 recommendations with grades ranging from B to D. The experts felt strongly that patients should be selected for CR based on the risk of structural damage, with routine CR of the hands and feet in rheumatoid factor-positive polyarticular JIA but not in oligoarticular non-extensive JIA.

**Conclusion:**

These first pragmatic recommendations on CR in JIA rely chiefly on expert opinion, given the dearth of scientific evidence. CR deserves to be viewed as a valuable tool in many situations in patients with JIA.

**Key Points:**

• *CR is a valuable imaging technique in selected indications.*

• *CR is routinely recommended for peripheral joints, when damage risk is high.*

• *CR is recommended according to the damage risk, depending on JIA subtype.*

• *CR is not the first-line technique for imaging of the axial skeleton.*

**Electronic supplementary material:**

The online version of this article (10.1007/s00330-018-5304-7) contains supplementary material, which is available to authorized users.

## Introduction

Juvenile idiopathic arthritis (JIA) is a heterogeneous group of chronic inflammatory joint conditions that can cause structural damage [1]. Seven mutually exclusive subtypes of JIA are defined in the 2001 Edmonton classification developed by the International League Against Rheumatism (ILAR) [2]. This classification has been challenged and modifications suggested, such as exclusion of systemic-onset JIA (sJIA) due to its similarity to autoinflammatory diseases [3, 4].

The prevalence of joint damage among patients with JIA has been estimated at 8–27 % in extended oligoarticular JIA (oJIA), 35–67 % in polyarticular JIA (pJIA) and up to 80 % in rheumatoid factor (RF)-positive pJIA [5, 6]. The main treatment objectives in JIA are to control the pain and to prevent structural damage. Joint space narrowing (JSN), bone erosions and demineralization are radiographic findings shared between JIA and adult rheumatoid arthritis (RA). Changes specific to the paediatric population are early growth plate closure, epiphyseal deformity and growth asymmetry [7].

Conventional radiography (CR), magnetic resonance imaging (MRI) and ultrasound (US) are the imaging modalities most often used to evaluate joint inflammation or structural damage [8]. MRI and US hold considerable promise but are still under evaluation in JIA. CR remains the most readily available imaging technique for detecting and monitoring structural damage. However, potential limitations of CR in JIA include the risk of radiation-induced harm to the patient, interpretation difficulties raised by skeletal immaturity, and the delayed development of structural joint damage. Furthermore, because JIA is rare, little is known about the potential effects of synthetic or biological disease-modifying anti-rheumatic drugs (DMARDs) on structural joint damage [9–11]. Thus, whereas recommendations based on large studies are available for the radiographic assessment of chronic inflammatory joint disease in adults [12, 13], no similar guidelines have been developed for JIA. A task force was recently convened by the European League Against Rheumatism (EULAR) – Paediatric Rheumatology European Society (PReS) to develop recommendations about imaging studies for diagnosing and managing JIA [14]. Although this undertaking acknowledged, for the first time, that an assessment of imaging studies in JIA was needed, the task force neither focussed on CR nor provided specific guidance for everyday practice.

We established a multidisciplinary task force to develop guidelines on the use of CR for the diagnosis and follow-up of each JIA subtype in everyday practice. Our project was supported by the French Society for Rheumatology (SFR), French Society for Paediatric Rheumatology and Internal Medicine (SOFREMIP), French Society for Paediatric and Prenatal Imaging (SFIPP), French Society for Radiology (SFR), and largest non-profit paediatric rheumatology patient organisation in France (KOURIR).

## Methods

### Field of research

We considered the following situations, at diagnosis and during follow-up, in each of the following five subtypes of JIA (oJIA, pJIA with and without RF and/or anti-citrullinated peptide antibody (ACPA), juvenile psoriatic arthritis (jPsA), and enthesitis-related arthritis (ERA)) Undifferentiated arthritis, as a heterogeneous subset related to one or several subtypes, and systemic JIA, having a peculiar articular course and structural prognosis, were left aside**.** Experts also focused on juvenile monoarthritis. Special attention was directed to the cervical spine, hip and temporo-mandibular joints (TMJs).

### Recommendation development process

The task force comprised 16 JIA experts (eight rheumatologists, five paediatricians, two paediatric radiologists experienced in skeletal disease and one patient organisation representative). We used the Grading of Recommendations, Assessment, Development and Evaluation (GRADE) method [15, 16] for elaborating, evaluating, disseminating and implementing recommendations elaborated by the EULAR and the Outcome Measures in Rheumatology (OMERACT) group [17, 18], and the Population, Intervention, Comparison, Outcome (PICO) process to frame the research questions.

We considered structural radiographic abnormalities: JSN, erosions, pseudo-joint space widening for sacro-iliac joint [19, 20] and ankylosis [12]. A research fellow (PM) assisted by two experts in systematic review methodology (CGV, methodologist; and VDP, convenor) performed a systematic literature review by searching PubMed, Scopus/Elsevier, and the Cochrane Library. Original articles including clinical trials, retrospective cohort studies, other retrospective studies, and case-control studies published between 1980 and December 2016 were identified. The following indexing was used: ‘juvenile idiopathic arthritis’ OR ‘juvenile rheumatoid arthritis’ OR ‘juvenile chronic arthritis’ OR ‘juvenile psoriatic arthritis’ OR ‘enthesitis-related arthritis’ OR ‘juvenile spondyloarthritis’ AND ‘radiography’ OR ‘X-ray’ (see Appendix 1 for details). The quality of evidence and grades of recommendation were determined according to the standards of the Oxford Centre for Evidence-Based Medicine [21]. Recommendations were graded A to D depending on the level of the underlying evidence (from 1A to 4) [18].

The task force debated and formulated a preliminary set of recommendations based on the systematic literature review supplemented, when necessary, by their expert opinion. This set was then evaluated by a panel of 14 independent French-speaking experts. Modifications were debated by the task force. The final recommendations were then rated on a 10-point scale by the task force and independent panel through a Delphi process.

## Results

### Systematic literature review

**Fig. 1.**
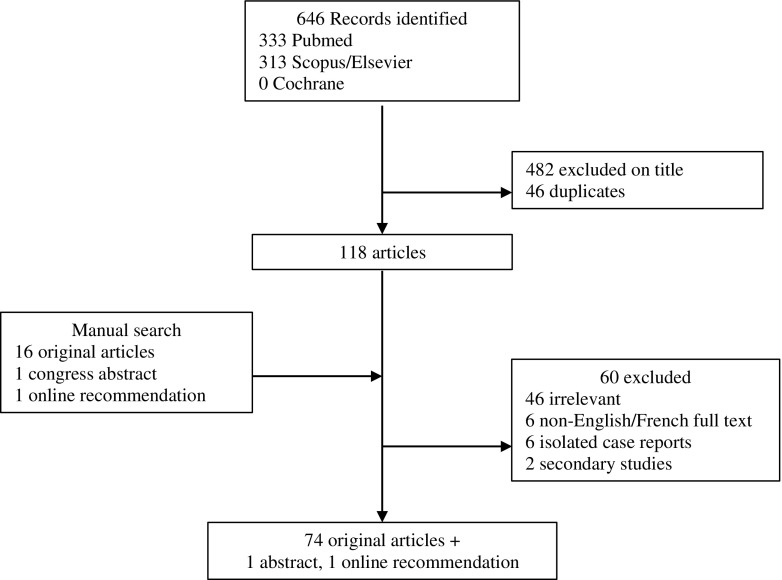
Systematic literature review flow-chart

Of the 118 publications identified by the literature search, 74 [5, 6, 9–11, 19, 20, 22–88] original articles, as well as one abstract [89] and one online recommendation [90], were included (Fig. 1, Table 1).

**Table 1:** Details of the studies identified by the systematic literature review

Article	Design	JIA subtype	Number of patients	Imaging findings used as outcome	Imaging technique	Purpose
Maldonado-Cocco 1980 [46]	Prospective	JRA	100	Primary	CR	To assess the frequency of carpal ankylosis
Williams and Ansell 1985 [54]	Retrospective	RF+ pJIA	81	Primary	CR	To assess peripheral radiographic progression
Poznanski 1991 [26]	Narrative review	JRA	NA	NA	CR	To develop a first score for assessing radiographic damage
Harel 1993 [10]	Prospective	JRA	23	Primary	CR	To assess effects of MTX on radiographic progression evaluated based on carpal length
Ravelli 1998 [11]	Retrospective	pJIA	26	Primary	CR	To assess carpal length changes during MTX therapy in pJIA (with bilateral wrist involvement)
Guillaume 2000 [35]	Prospective	oJIA	207	Secondary	CR	To identify prognostic factors in oJIA
Al-Matar 2002 [36]	Retrospective	oJIA	205	Secondary	CR	To identify early features associated with poor outcome in oligoarticular-onset JIA
Flatø 2002 [30]	Retrospective	JRA, SEA, JPsA, IBD- associated arthritis	314	Primary	CR	To assess factors associated with radiographic sacroiliitis in JIA
Huemer 2002 [64]	Prospective	JPsA, oJIA	87	No	NA	To compare clinical features of JPsA and oJIA, including patterns of joint involvement, and to discuss classification
Laiho 2002 [70]	Cross-sectional	JCA	159	Primary	CR	To evaluate radiographic inflammatory changes in the cervical spine
Mason 2002 [49]	Cross-sectional	Polyarticular JRA	60	Primary	CR	To assess the frequency of in hand/wrist CR damage at diagnosis
Oen 2002 [39]	Narrative review	JIA	NA	NA	NA	To identify outcome predictors, including radiographic findings
Bowyer 2003 [40]	Retrospective	oJIA, pJIA, sJIA	703	Secondary	CR	To assess health status 1 and 5 years after disease onset
Doria 2003 [45]	Cross-sectional	JRA	60	Primary	CR	To assess inter- and intra- observer variability of two scoring systems (Larsen/modified Larsen), comparison to MRI
Flatø 2003 [50]	Case-control	JRA	268	Secondary	CR	To assess long-term prognostic factors
Magni-Manzoni 2003 [51]	Prospective	pJIA, extended oJIA, sJIA, JPsA, ERA	94	Primary	CR	To assess the rate of radiographic progression (Poznanski score)
Oen 2003 [38]	Retrospective	JRA	216	Primary	CR	To assess radiographic damage in early and advanced disease
Oen 2003 [37]	Retrospective	JRA	393	Secondary	CR	To identify early predictors of long-term outcome
Ravelli and Martini 2003 [6]	Narrative review	All subtypes	NA	NA	NA	To identify early predictors of outcomes, including radiographic outcomes
Tsitsami 2003 [68]	Retrospective	oJIA, JPsA, UA	185	Secondary	CR	To evaluate associations between a familial history of psoriasis and the outcome of oligoarticular JIA
Van Rossum 2003 [31]	Prospective	pJIA, oJIA, extended oJIA	67	Primary	CR	To describe radiographic features
Twilt 2004 [73]	Cross-sectional	JIA (all subtypes)	97	Primary	CR	To evaluate the prevalence of radiographic damage on the OPG
Mason 2005 [5]	Prospective	Polyarticular JRA	12	Primary	CR	To assess radiographic progression after 2 years
Van Rossum 2005 [29]	Prospective	pJIA, oJIA	66	Primary	CR	To assess sensitivity of Dijkstra radiographic score
Helenius 2006 [83]	Prospective	Adult: RA, AS, SPA, MCTD	67	Primary	CR, MRI	To describe clinical, radiographic and MRI findings in rheumatic diseases
Rossi 2006 [33]	Prospective	pJIA	25	Primary	CR	To assess the reliability of the Sharp and Larsen radiographic scoring systems
Flatø 2006 [58]	Case / control	ERA/oJIA, pJIA	55/55	Secondary	CR	To compare clinical, functional and radiological features in ERA versus other JIA subtypes
Selvaag 2006 [28]	Prospective	sJIA, pJIA, oJIA, ERA	137	Primary	CR	To assess radiographic findings at diagnosis and 3-years later
Billiau 2007 [82]	Prospective	sJIA, RF+ and RF- pJIA, oJIA, ERA, JPsA	100	Secondary	CR	To describe clinical, orthodontic, OPG and lateral cephalogram in 46 patients
Gilliam 2008 [44]	Retrospective	RF+ and RF- pJIA, oJIA, sJIA	68	Secondary	CR	To evaluate associations of markers, including radiographic changes, to disease severity
Habib 2008 [47]	Cross-sectional	pJIA, sJIA, oJIA	68	Secondary	CR	To determine the prevalence and significance of ACPAs in JIA
Nielsen 2008 [9]	Retrospective	extended oJIA, sJIA, pJIA, JPsA	40	Primary	CR	To evaluate the radiographic outcome (Poznanski score) during etanercept therapy
Pedersen 2008 [84]	Prospective	JIA (subtype not specified)	15	Primary	CR, MRI	To describe clinical, CRand MRI features; to compare CR to MRI
Rostom 2008 [57]	Cross-sectional	JIA (all subtypes)	121	Primary	CR	To determine the prevalence of clinical and radiological hip involvement
Müller 2009 [77]	Prospective	JIA (all subtypes)	30	Primary	US, MRI	To compare clinical examination/US to MRI
Butbul 2009 [62]	Retrospective	JPsA, oJIA, pJIA	106	No	NA	To compare clinical features in JPsA to other JIA subtypes with similar patterns of joint disease – including growth abnormalities
Endén 2009 [71]	Cross-sectional	sJIA, pJIA/ fibromyalgia (control)	134/24	Primary	CR	To describe growth and cervical vertebrae size in JIA (vs. control)
Flatø 2009 [63]	Retrospective	JPsA, oJIA, pJIA	336	Secondary	CR	To compare JPsA features (including radiographic sacro-iliitis) and outcomes to other JIA subtypes
Lin 2009 [60]	Cross-sectional	Juvenile AS	47 juvenile AS, 122 adult AS	Secondary	CR	To compare clinical, laboratory and radiographic features between juvenile and adult-onset AS
Tafaghodi 2009 [34]	Retrospective	JIA (all subtypes)	174	Primary	CR	To assess radiographic characteristics of JIA (118 patients) vs. ALL (56 patients)
Arvidsson 2010 [76]	Prospective	JRA	60	Primary	CR, CT	To assess TMJ imaging during follow-up for long-standing JIA
Pagnini 2010 [20]	Prospective	ERA	59	Primary	CR, MRI	To identify predictors of sacroiliitis
Stoll 2010 [19]	Retrospective	ERA, JSpA, JPsA	143	Primary	CR, MRI	To identify risk factors for sacroiliitis
Cannizzaro 2011 [85]	Retrospective	oJIA, RF+ and RF- pJIA, JPsA, ERA, sJIA	223	Secondary	CR, MRI	To determine the incidence of TMJ involvement in different JIA subtypes
Kjellberg 2011 [72]	Case-control	pJIA, oJIA, JPsA, ERA, UA	82	Primary	CR	To compare radiographic cephalometry findings in JIA and healthy controls
Ravelli 2011 [24]	Retrospective	oJIA, RF- negative pJIA, JPsA, UA	971	Secondary	CR	To compare disease characteristics depending on ANA status
Stoll 2011 [65]	Retrospective	JPsA oJIA	87/303	No	NA	To compare clinical features of oJIA vs. JPsA
Stoll 2011 [66]	Narrative review	JPsA	NA	No	NA	To identify features of JPsA, in comparison with other subtypes of JIA
Bertilsson 2012 [41]	Prospective	JCA	132	Secondary	CR	To prospectively investigate the characteristics and outcome predictors over 5 years of follow-up
Lipinska 2012 [27]	Prospective	oJIA, pJIA, sJIA	74	Secondary	CR	To assess the Steinbrocker score depending on ACPA status
Bertilsson 2013 [42]	Prospective	JCA	132	Secondary	CR	To evaluate long-term outcomes, after 17 years of follow-up
Chen 2012 [56]	Cross-sectional	Juvenile- onset AS	67	Secondary	CR	To compare clinical, laboratory and radiographic features of juvenile-/adult-/late-onset AS
Ozawa 2012 [52]	Cross-sectional	pJIA, sJIA	40	Secondary	CR	To compare radiological and laboratory findings in pJIA and sJIA
Abramowicz 2013 [75]	Retrospective	JIA	51	Primary	MRI	To identify prevalence of synovitis on MRI, TMJ imagingand clinical predictive factors
Elhai 2013 [48]	Prospective	pJIA	43	Primary	CR	To compare radiological outcomes of pJIA at transition vs. matched RA patients
Elhai 2013 [69]	Cross-sectional	pJIA/RA	57/58	Primary	CR	To compare the frequency of cervical spine radiographic damage between long-standing pJIA and RA
Jadon 2013 [59]	Systematic review	Juvenile-onset AS	NA	NA	CR	To compare clinical, social and radiographic features of adult- vs. juvenile-onset AS
Omar 2013 [53]	Cross-sectional	oJIA, pJIA, sJIA	54	Secondary	CR	To assess correlations linking ACPA presence to the JADAS and Sharp van der Heijde scores
Cedströmer 2013[78]	Retrospective	oJIA, sJIA, pJIA, JPsA, ERA	266	Secondary	CR	To describe clinical findings and disease activity and their associations with CR abnormalities
Giancane 2014 [43]	Prospective	RF+ and RF- pJIA, sJIA, extended oJIA, UA, JPsA	186	Primary	CR	To assess radiographic outcomes during follow-up (1–10 years)
Jaremko 2014 [61]	Cross-sectional	Juvenile AS	26	Primary	CR, MRI	To compare the usefulness of CR and MRI for sacro-iliac joint evaluation at diagnosis of juvenile AS
Rodriguez-Lozano 2014 [32]	Cross-sectional	sJIA, RF+ and RF- pJIA, JPsA, extended oJIA	60 CR	NA	CR	To assess the inter-observer reliability of CR interpretation
Abramowicz 2014 [74]	Retrospective	oJIA, pJIA, JPsA	30	Primary	CR, MRI	To identify radiographic findings associated with TMJ synovitis on MRI
Górska 2014 [79]	Cross-sectional	oJIA, pJIA	26	Primary	CR	To describe orthodontic and radiographic findings
Koos 2014 [80]	Case-control	oJIA, RF- negative pJIA, ERA, JPsA/non-JIA controls	23/23	Primary	Cone Beam CT	To describe pathological changes in TMJs
Koos 2014[81]	Cross-sectional	JIA (all subtypes)/controls	134/134	Primary	MRI	To evaluate the reliability of clinical symptoms for diagnosing TMJ synovitis
Ringold 2014 [55]	Recommendations	pJIA	NA	NA	CR	To develop CARRA recommendations for treating new-onset pJIA
Colebatch-Bourn 2015 [87]	Recommendations	All subtypes	NA	NA	CR, US, MRI	EULAR recommendations/ all imaging techniques
Ravelli 2015 [23]	Narrative review	JPsA	NA	NA	No	To assess the classification of JPsA and its relation to oJIA
Chan 2016 [22]	Prospective	JPsA and non-psoriatic JIA	57	No	No	To discuss the classification of JPsA
Jadon 2016 [88]	Prospective	Adult AS and PsA	402	Primary	CR	To compare radiographic features of AS vs. PsA with axial disease
Kavanaugh 2016[25]	Phase III clinical trial	Adult PsA	405	Primary	CR	To assess the efficacy of golimumab on radiographic progression in adult PsA
Kristensen 2016 [86]	Systematic review	All subtypes	NA	NA	MRI	To identify clinical predictors of TMJ involvement, needing imaging assessment
Weiss 2016 [67]	Prospective	JSpA	40	Primary	CR, MRI	To evaluate the prevalence of sacroiliitis, compared to physical examination findings
Guide du bon usage des examens d’imagerie (French online recommendation) [90]	Recommendations	NA	NA	NA	CR	To develop recommendations about CR for focal limb pain
Ravelli 2014 [89] (ACR Pediatric Rheumatology Symposium)	Clinical trial	pJIA	87	Primary	CR	To assess the effect of tocilizumab on pJIA after 2 years, using the van der Heijde and Poznanski scores

*ACPA* anti-citrullinated protein antibody, *ALL* acute lymphoblastic leukaemia, *ANA* antinuclear antibody, *AS* ankylosing spondylitis, *CARRA* Childhood Arthritis and Rheumatology Research Alliance, *CR* conventional radiography, *IBD* inflammatory bowel disease, *JADAS* Juvenile Arthritis Disease Activity Score, *JCA* juvenile chronic arthritis (former EULAR criteria), *JRA* juvenile rheumatoid arthritis (former ACR criteria), *JPsA* juvenile psoriatic arthritis, *JSpA* juvenile spondyloarthritis, *MCTD* mixed connective tissue disease, *MTX* methotrexate, *NA* not applicable, *oJIA* oligoarticular juvenile idiopathic arthritis, *OPG* orthopantomogram, *pJIA* polyarticular juvenile idiopathic arthritis, *SEA* seronegative enthesopathy and arthropathy, *sJIA* systemic juvenile idiopathic arthritis, *UA* undifferentiated arthritis

### Recommendations

The experts elaborated four overarching principles and 31 recommendations. Table 2 lists the recommendations.

**Table 2:** Recommendations about CR as a diagnostic and follow-up investigation in non-systemic JIA, with scores for agreement among experts, levels of evidence and grade

Recommendations	Mean agreement score (±SD)	Level of evidence	Grade
*Overarching principles*			
A. A CR assessment is necessary in JIA.	9.30 (±1.26)	-	-
B. The potential risks associated with exposure to ionising radiation must always be considered when using CR.	9.70 (±0.70)	-	-
C. CR is difficult to interpret in skeletally immature patients, particularly those <5 years of age.	8.95 (±1.73)	-	-
D. Other imaging techniques, such as US and MRI, are being developed in JIA, and will be discussed in specific recommendations.	8.95 (±1.85)	-	-
*Oligoarthritis (oJIA)*			
1. CR should not be performed routinely as a diagnostic investigation.	8.20 (±1.94)	3	C
2 During follow-up, CR should be performed on affected joint(s) that remain symptomatic* after 3 months	9.10 (±2.17)	4	D
3. In patients with persistently symptomatic* joints, the reiteration of CR during follow-up is at the discretion of the physician.	9.1 5(±1.04)	4	D
4. In patients with inactive disease, CR is not recommended.	9.45 (±0.83)	4	D
5. In patients with extended oJIA, the recommendations for pJIA should be applied.	9.30 (±0.92)	3	C
6. In patients with structural damage, the selection and timing of specific imaging techniques to further assess the damaged joint during follow-up is guided by clinical considerations.	9.15 (±1.04)	4	D
*Polyarthritis (pJIA)*			
7. Routine CR of the wrists, hands, and forefeet is strongly recommended at the diagnosis of polyarticular JIA with positive RF/ACPA.	9.30 (±1.26)	2B, 3	B
8. CR of other joints than wrists, hands, and forefeet, is recommended at the diagnosis for symptomatic* joints only.	9.00 (±1.49)	2B,3	B
9. In new-onset RF/ACPA-negative pJIA with adverse prognostic factors, CR at diagnosis should be performed as for RF/ACPA-positive pJIA (recommendation #7).	8.55 (±2.46)	3	C
10. Adverse prognostic factors are early wrist involvement, distal involvement, symmetric arthritis, high CRP/ESR, and bone erosions.	9.35 (±0.81)	2B	B
11. In new-onset, RF/ACPA-negative pJIA without adverse prognostic factors, at diagnosis, CR should be confined to symptomatic* joints.	8.15 (±2.28)	4	D
12. In RF/ACPA-positive pJIA, CR of the hands, wrists, and forefeet is strongly recommended	8.6 (±1.31)	2A, 2B	B
- 1 year after disease onset	8.30 (±1.72)	2B	B
- and when transitioning from paediatric to adult healthcare	8.85 (±0.99)	4	D
At other time points, the use of CR during follow-up is at the discretion of the physician.	9.25 (±0.85)	4	D
13. Routine CR of other joints is not recommended.	9.40 (±0.75)	4	D
14. During the follow-up of RF/ACPA-negative pJIA with adverse prognostic factors, CR should be performed as for RF/ACPA-positive pJIA (recommendation #12).	9.00 (±2.03)	3	C
15. During the follow-up of RF/ACPA-negative pJIA without adverse prognostic factors, the use of CR is at the discretion of the physician.	9.50 (±1.17)	4	D
16. CR can be repeated in patients who remain symptomatic longer than 3 months.	8.25 (±2.10)	4	D
17. In patients with structural damage, the selection and timing of specific imaging techniques during follow-up is guided by clinical considerations.	9.35 (±0.81)	4	D
*Enthesitis-related arthritis (ERA)*			
18. In patients with axial ERA, CR of the spine and hip joints should be performed only when needed for the differential diagnosis.	8.05 (±2.42)	4	D
19. During the follow-up of axial ERA, CR should be considered only for the hip joints, depending on the clinical course and availability of US and/or MRI.	8.90 (±1.33)	3	C
20. CR is not recommended for multifocal enthesitis.	9.10 (±0.97)	4	D
21. In patients with isolated enthesitis, CR can be considered as a tool for establishing the differential diagnosis.	8.35 (±2.43)	4	D
*Psoriatic arthritis (jPsA)*			
22. No specific recommendation can be made about CR in juvenile psoriatic arthritis.	9.20 (±0.83)	4	D
23. Guidance may be taken from the recommendations above, depending on the clinical presentation, or from recommendations issued for adults.	9.35 (±0.74)	4	D
*Situations of specific interest*			
*Monoarthritis*			
24. At the diagnosis of acute monoarthritis, CR of the involved joint should be performed, with two perpendicular views.	9.35 (±1.04)	3	C
25. At the diagnosis of acute monoarthritis, comparative CR of the contralateral joint is unnecessary.	8.50 (±2.39)	4	D
26. In patients with persistent neck pain related to JIA, MRI is preferable over CR.	9.60 (±0.68)	4	D
27. When MRI is unavailable, CR is recommended only for the cervical spine and should consist only in a lateral view.	8.80 (±1.56)	4	D
28. In patients with JIA who have neurological symptoms of spinal cord compression and neck pain, cervical MRI must be performed, on an emergency basis.	9.80 (±0.52)	3	C
29. CR of the TMJs is not recommended when cross-sectional imaging is available.	9.20 (±1.47)	3	C
30. Routine CR of the hip joint is not recommended in patients with pJIA.	9.25 (±1.02)	3	C
31. When CR of a symptomatic hip joint is performed, a single view should be obtained, i.e., either an antero-posterior view or a frog leg view.	9.05 (±1.28)	4	D

*JIA* juvenile idiopathic arthritis, *CR* conventional radiography, *oJIA* oligoarticular juvenile idiopathic arthritis, *pJIA* polyarticular juvenile idiopathic arthritis, *RF* rheumatoid factor, *ACPA* anti-citrullinated protein antibody, *ERA* enthesitis-related arthritis, *TMJ* temporo-mandibular joint

*Symptomatic joints: swollen and/or painful joints, and/or joints with motion range limitation

### Overarching principles

Radiation exposure was taken into account (principle B), according to French Society for Radiology recommendations [90] (Appendix 2). Much of the cartilage is still radio-transparent in children younger than 5 years of age. In this age group, the need for CR must be evaluated with great care (principle C) [91].

Other imaging modalities such as US and MRI are increasingly used in JIA. Although promising, they are not discussed herein. They will be the focus of specific recommendations (principle D).

#### Oligoarticular JIA (oJIA)

1. *CR should not be performed routinely as a diagnostic investigation in oJIA*. The literature review identified ten studies in which CR was performed, even in patients younger than 4 years. Among them, one focussed specifically on oJIA [35] and nine investigated several JIA subtypes but reported data separately for oJIA [6, 24, 27, 36–38, 40, 42, 43]. The usefulness of CR is limited by the incomplete ossification of the epiphyses, most notably in the youngest age groups [33]. Therefore, when the diagnosis is definitive, CR is not recommended.

2. and 3. During follow-up, CR should be performed on affected joint(s) that remain symptomatic after 3 months. By ‘symptomatic joints’*, we mean painful and/or swollen joints and/or joints that are limited in motion. In patients with persistently symptomatic* joints, the reiteration of CR during follow-up is at the discretion of the physician. Several studies showed evidence of radiographic progression early in the natural history of oJIA [24, 27, 35, 38].

4. *In patients with clinically inactive disease (CID), CR should not be performed routinely*. The diagnosis of CID relies on physician judgement, aided by validated tools [92–94]. No data are available on radiographic disease progression in clinically silent joints in patients with oJIA.

5. *In patients with extended oJIA, the recommendations for pJIA should be applied.* The number of affected joints is strongly associated with structural damage in oJIA [35].

6. *In patients with structural damage, the selection and timing of specific imaging techniques to further assess the damaged joint during follow-up is guided by clinical considerations.*

Joints with structural damage must undergo specific CR evaluations during the patient’s growth.

#### Polyarticular JIA (pJIA)

7. and 8. Routine CR of the wrists, hands and forefeet is strongly recommended at the diagnosis of polyarticular JIA with positive RF/ACPA. CR of other joints than wrists, hands and forefeet, is recommended at the diagnosis for symptomatic joints*only. Prospective studies were reviewed, with special attention to early pJIA. Erosions and JSN occurred preferentially at the hands, wrists and feet [11, 31, 43, 48–51], joints that were sometimes asymptomatic [31] CR at the diagnosis provides a reference for assessing disease progression. It is supported by ‘adult’ recommendations [13] for rheumatoid arthritis, which has a similar structural evolution.

9. and 10. *In new-onset RF/ACPA-negative pJIA with adverse prognostic factors, CR at diagnosis should be performed as for RF/ACPA-positive pJIA.* Box 1 lists the factors of adverse prognostic significance in pJIA [31, 44, 50, 51]. These factors are associated with a pattern of joint damage over time similar to that seen in RF/ACPA-positive pJIA [38].

Box 1: Factors of adverse prognostic significance in polyarticular juvenile idiopathic arthritis (pJIA)

**Table:** 

Early involvement of wrists Symmetric arthritis Distal, small-joint arthritis Elevated ESR/CRP Pre-existing radiographic abnormalities	

ESR, erthrocyte sedimentation rate; CRP, serum C-reactive protein level

11. In new-onset, RF/ACPA-negative pJIA without adverse prognostic factors, at diagnosis, CR should be confined to symptomatic* joints. This recommendation is based on expert opinion.

12. *In RF/ACPA-positive pJIA, CR of the hands, wrists and forefeet is strongly recommended 1 year after disease onset, and when transitioning from paediatric to adult healthcare. At other time points, the use of CR during follow-up is at the discretion of the physician.* Prospective studies found evidence of joint damage even in asymptomatic joints [31]. Patients with long-standing disease had high prevalences of joint erosions (30–70 % in historical studies) [5, 28, 38, 40, 44, 48, 54], close to those in adults with RA [48]. In RA, joint destruction at asymptomatic sites is a major predictor of adverse outcomes [13, 95]. However, radiographic progression with erosions in asymptomatic joints is not well documented in JIA and may have been underestimated. In a study of 471 joints in 67 patients with polyarticular JIA, radiographs showed erosions at the hands and feet in 36 % and 39 % of cases, respectively [31]. Our literature review identified some data on the best times for CR. One study suggested a higher risk of radiographic progression within the first year after disease onset [51]. The experts felt that CR contributed to ease the transition from paediatric to adult healthcare [96].

13. *Routine CR of other joints is not recommended***.** No data were found on which to base specific recommendations.

14. *During the follow-up of RF/ACPA-negative pJIA with adverse prognostic factors, CR should be performed as for RF/ACPA-positive pJIA* (see recommendation #12).

15. *During the follow-up of RF/ACPA-negative pJIA without adverse prognostic factors, the use of CR is at the discretion of the physician****.*** No scientific data were available on which to base specific recommendations.

16. and 17. CR can be repeated in patients who remain symptomatic* longer than 3 months. In patients with structural damage, the selection and timing of specific imaging techniques during follow-up is guided by clinical considerations. The experts emphasised the need for careful attention to joints with active disease. In prospective studies, the time interval separating CR assessments of the same joints ranged from 8 months to 24 years. The 3-month interval in this recommendation was based on expert opinion.

#### Enthesitis-related arthritis (ERA)

18. In patients with axial ERA, CR of the spine and hip joints should be performed only when needed for the differential diagnosis. Axial manifestations may arise at the spine, hips and sacro-iliac joints. A radiographic view specifically designed to assess the sacro-iliac joints is not recommended, as the results are not interpretable in skeletally immature patients and radiation exposure is significant [20]. In patients with axial inflammatory pain, MRI (for both sacro-iliac and hip joints) and US (for the hip joint) may be more relevant [67].

19. *During the follow-up of axial ERA, CR should be considered only for the hip joints, depending on the clinical course and availability of US and/or MRI*. ERA is associated with a high prevalence of hip joint arthritis [30, 56, 58–60]. MRI or US are non-irradiating methods capable of detecting hip joint effusion; in addition, MRI can detect bone oedema. Therefore, in the future, MRI and US may deserve consideration as first-line imaging techniques. CR, however, is appropriate for monitoring known structural damage and deformities.

20. and 21. *CR is not recommended for multifocal enthesitis. In patients with isolated enthesitis, CR can be considered as a tool for establishing the differential diagnosis.* When isolated enthesitis is suspected, CR may contribute to the differential diagnosis (e.g. with post-traumatic changes or osteochondritis); otherwise, CR is unhelpful for assessing peri-articular manifestations.

#### Psoriatic juvenile arthritis (jPsA)

22. *No specific recommendation can be made about CR in juvenile psoriatic arthritis.* Scientific data are scarce [62–66, 68]. The definition of this entity is still debated [68]. Traditionally, two subtypes are described, an axial inflammatory disease resembling axial ERA and a peripheral joint disease resembling oJIA [66].

23. Guidance may be taken from the recommendations above, depending on the clinical presentation, or from recommendations issued for adults.

#### Situations of specific interest

##### Monoarthritis

24. *At the diagnosis of acute monoarthritis, CR of the involved joint should be performed, with two perpendicular views*. The French Society for Radiology [90] strongly recommends CR of any site of focal bone pain in paediatric patients, with the goal of excluding a tumour, osteomyelitis, or a haematological malignancy [34, 97].

25. *At the diagnosis of acute monoarthritis, comparative CR of the contralateral joint is unnecessary*. Because cartilage thickness varies within individuals, comparison to the healthy contra-lateral joint is uninformative [26, 33].

##### Cervical spine

26. *In patients with persistent neck pain related to JIA, MRI is preferable over CR*.

27. *When MRI is unavailable, CR is recommended only for the cervical spine and should consist only of a lateral view.*

28. *In patients with JIA who have neurological symptoms of spinal cord compression and neck pain, cervical MRI must be performed, on an emergency basis*.

In a cohort study of oJIA, 2.4 % of patients had cervical spine damage at the diagnosis [35]. Cervical spine erosions and ankylosis are common in advanced pJIA [42, 71]. Evidence-based data are too scarce to recommend any specific pattern of radiological follow-up. Atlanto-axial diastasis may be normal in paediatric patients, and dynamic CR is therefore irrelevant. MRI is the most sensitive imaging technique, and is mandatory when spinal cord compression is suspected [98].

##### Temporomandibular joints

29. *CR of the TMJs is not recommended when cross-sectional imaging is available*.

TMJ damage is common in JIA, with the prevalence ranging across studies from 17 % to 87 % [73]. The TMJ cartilage is thin and condylar erosions therefore develop early. The panoramic radiograph is often normal at disease onset. Cross-sectional imaging offers better diagnostic performance. Imaging of the TMJs is not usually performed on a routine basis but is required in the event of pain, mouth-opening limitation or audible cracking of the TMJs [74, 76–81, 83, 84]. MRI is considered the best imaging technique, although distinguishing the normal appearance from abnormal changes can be challenging [99, 100]. Cone-beam computed tomography allows three-dimensional reconstructions [101]. The usefulness of US TMJ imaging is under debate [77, 102].

##### Hip joint

30. *Routine CR of the hip joint is not recommended in patients with pJIA.*

31. *When CR of a symptomatic hip joint is performed, a single view should be obtained, i.e. either an antero-posterior view or a frog leg view.*

In RF/ACPA-positive pJIA, hip joint damage is common [48] but CR of the hip joint is associated with a high level of ionising radiation exposure, so the hip is not among the joints for which routine CR is recommended .When available, MRI should be performed instead of, or in addition to, CR. If CR is performed, either an antero-posterior or a frog leg view is recommended, to visualise both hip joints and to allow the detection of bone erosions and/or avascular necrosis.

## Discussion

CR is the most widely available imaging procedure worldwide. In paediatric patients, this advantage should be weighed against the heightened risks of radiation exposure and difficulty in interpreting joint radiographs before skeletal maturity is achieved. In addition, in JIA, radiographically visible joint damage takes time to develop, limiting the usefulness of CR. Specific recommendations about CR in paediatric patients are therefore needed, a fact that prompted the present work.

Obstacles to the development of recommendations about CR in JIA included the paucity of strong evidence about structural disease progression in JIA and the pooling of JIA subtypes in many studies. The low incidence of JIA contributes to explain the dearth of data. To maximise the usefulness of our recommendations to all physicians caring for patients with JIA, we focussed on CR and separated the five non-systemic, non-undifferentiated subtypes of JIA. Importantly, these recommendations are based not only on recently published data, but also, in many cases, on expert opinion, due to the paucity of paediatric studies. As a result, many of our recommendations are low grade, and in some cases obtaining guidance from recommendations for adults would seem to be the only option. However, the level of agreement among the multidisciplinary experts sitting on our panel was high.

Structural damage requires evaluation in JIA, especially in pJIA and extended oJIA, which carry the highest risk of adverse outcomes. In the treatment plans for pJIA developed by the CARRA, CR changes are considered an important outcome and their yearly assessment is suggested [55]. However, the risk associated with exposure to ionising radiation during CR is of major concern, as pointed out by the representative of the patient organisation during our study. Little evidence is available on which to base an objective quantification of this risk. Our experts considered that the risk was substantial for CR of the pelvis and lumbar spine but was too small at peripheral sites to constitute an argument against using CR. To minimise radiation exposure, the experts recommended having CR performed at centres with expertise in paediatric radioprotection.

Research is needed in a broad range of areas to fill the knowledge gaps we identified when developing our recommendations (Box 2). More specifically, most paediatric clinical trials failed to assess potential treatment effects on structural damage. Also, data on structural damage just before the transition to adult healthcare are needed, since treatment recommendations for adults are based on structural damage.

Box 2: Research agenda

**Table:** 

- Follow-up of a cohort of patients with recent-onset RF/ACPA-positive polyarticular JIA, with annual CR for 10 years to identify predictors of structural joint damage - Comparison of radiographic disease progression in oligoarticular JIA in patients with and without antinuclear antibodies - Comparison of joint MRI, US, and CR as tools for detecting structural damage in patients younger than 5 years of age - Evaluation of joint damage at the transition from paediatric to adult healthcare in each JIA subtype - Improvement of the definition of juvenile psoriatic arthritis, to obtain homogeneous populations for studies of imaging techniques	

We considered neither MRI nor US, both of which are under evaluation in JIA. Both are non-irradiating, and US is also widely available and inexpensive, although it requires specific training. US is now performed almost routinely in adults with joint disease. In paediatric patients, however, differentiating normal from abnormal findings by MRI and US can be challenging [100, 103]. Furthermore, very few physicians are specifically trained in paediatric US. The OMERACT and Health-e-Child Radiology groups are currently working together to standardise MRI protocols and interpretation in JIA [104–106].

In conclusion, CR still appears relevant in many situations in patients with JIA. CR is a widely available and inexpensive investigation that has an acceptable safety profile and can provide essential information about the structural course of the disease. Until validation studies of other imaging techniques, such as MRI and US, are completed, CR will remain the investigation of reference for assessing structural joint damage in patients with JIA.

## Electronic supplementary material

Appendix 1 (DOCX 19 kb)

Appendix 2 (DOC 36 kb)

## Abbreviations

ACPA: Anti-Citrullinated Protein Antibody
CR: Conventional radiography
DMARDs: Disease-modifying Antirheumatic drugs
ERA: Enthesitis-related arthritis
EULAR: European League Against Rheumatism
GRADE: Grading of Recommendations, Assessment, Development and Evaluation
ILAR: International League Against Rheumatism
JIA: Juvenile idiopathic arthritis
jPsA: Juvenile psoriatic arthritis
JSN: Joint space narrowing
MRI: Magnetic resonance imaging
oJIA: Oligoarticular juvenile idiopathic arthritis
OMERACT: Outcome Measures in Rheumatology
PReS: Paediatric Rheumatology European Society
PICO: Population, Intervention, Comparison, Outcome
pJIA: Polyarticular juvenile idiopathic arthritis
RA: Rheumatoid arthritis
RF: Rheumatoid factor
SFIPP: French Society for Paediatric and Prenatal Imaging
SFR: French Society for Radiology
SFR: French Society for Rheumatology
sJIA: Systemic juvenile idiopathic arthritis
SLR: Systematic literature review
SOFREMIP: French Society for Paediatric Rheumatology and Internal Medicine
TMJ: Temporo-mandibular joint
US: Ultrasound
