# Supplementary material for: Conventional radiography in juvenile idiopathic arthritis: Joint recommendations from the French societies for rheumatology, radiology and paediatric rheumatology
Source: Eur Radiol. 2018 Mar 26;28(9):3963–76. doi: 10.1007/s00330-018-5304-7 (PMC6096609; doi:10.1007/s00330-018-5304-7)
Supplement: Supplementary file 1 — (DOCX 19 kb) [file 330_2018_5304_MOESM1_ESM.docx]

**APPENDIX 1**

A systematic literature review was performed by a junior fellow (P-M) by searching PubMed, Scopus/Elsevier, and the Cochrane Library using the following indexing: “juvenile idiopathic arthritis” OR “juvenile rheumatoid arthritis” OR ”juvenile chronic arthritis“ OR “juvenile psoriatic arthritis” OR “enthesitis-related arthritis” OR “juvenile spondyloarthritis” AND “radiography” OR “X-ray”. This search identified 333 publications on PubMed, 313 on Scopus/Elsevier, and none in the Cochrane Library. Selecting potentially relevant records based on the title identified 118 articles on PubMed, as well as 46 on Scopus/Elsevier, all of which were also in the PubMed group. Given the scarcity of data on conventional radiography in juvenile idiopathic arthritis (JIA), we did not select articles based on methodology or level of evidence. The paucity of data is illustrated by the finding of only four PubMed articles using “juvenile psoriatic arthritis” AND “radiography”. We included all studies published in English between 1980 and December 2016 and reporting retrospective or prospective cohort studies of JIA patients with data on radiographic progression (usually as a secondary endpoint). Manually searching the reference lists of selected articles using key words of greater specificity (e.g., “joint space narrowing” and “erosions”) retrieved 15 additional articles. We also looked at one abstract presented at a meeting [93], online recommendations issued by scientific societies [94], and European legislation [19] **(table 2).**

A vast majority of studies identified by our search were conducted before the advent of biologics. Radiographic endpoints were rarely considered in clinical trials of non-systemic JIA; the efficacy of etanercept on the Poznanski score was assessed in one study [8]. Radiographic damage is probably less common now than in the pre-biologics era. Another difficulty resided in identifying the JIA subtypes in studied patients. More specifically, “polyarticular JIA” was often used as a term encompassing sJIA, RF-positive pJIA, RF-negative pJIA, and extended oJIA. Finally, no articles on radiographic progression in psoriatic JIA were available, and we supplemented this deficiency by looking at data from adult cohorts or patients with JIA presentations resembling psoriatic JIA.

1. A11: Assessment of Radiographic Progression in Patients With Polyarticular-Course Juvenile Idiopathic Arthritis Treated With Tocilizumab: 2-Year Data From CHERISH - Ravelli - 2014 - Arthritis & Rheumatology - Wiley Online Library. http://onlinelibrary.wiley.com/enhanced/doi/10.1002/art.38422. Accessed 19 May 2016

2. Guide du bon usage des examens d’imagerie médicale. http://www.sfrnet.org/sfr/professionnels/5-referentiels-bonnes-pratiques/guides/guide-bon-usage-examens-imagerie-medicale/index.phtml. Accessed 30 Mar 2016

3. EUR-Lex - 32013L0059 - EN - EUR-Lex. http://eur-lex.europa.eu/legal-content/FR/TXT/?uri=CELEX%3A32013L0059. Accessed 27 Feb 2016

4. Nielsen S, Ruperto N, Gerloni V, et al (2008) Preliminary evidence that etanercept may reduce radiographic progression in juvenile idiopathic arthritis. Clin Exp Rheumatol 26:688–692.
