# Supplementary material for: Conventional radiography in juvenile idiopathic arthritis: Joint recommendations from the French societies for rheumatology, radiology and paediatric rheumatology
Source: Eur Radiol. 2018 Mar 26;28(9):3963–76. doi: 10.1007/s00330-018-5304-7 (PMC6096609; doi:10.1007/s00330-018-5304-7)
Supplement: Supplementary file 2 — (DOC 36 kb) [file 330_2018_5304_MOESM2_ESM.doc]

**Appendix 2 irradiation level**

A major issue that required discussion was the risk of radiation exposure in paediatric patients. This discussion was based on a literature review and European recommendations [1, 2]. The directive 2013/59 EURATOM strongly recommends caution when using CR and the preferential use of non-irradiating techniques when available and relevant [3]. Radioprotection involves both confining CR to situations where it is absolutely necessary and optimising the equipment and technique to ensure that the radiation dose is as low as reasonably achievable (ALARA). Little is known about the risk of repeated CR in paediatric patients. Historical studies in adults showed a higher risk for cancer in populations repeatedly exposed to radiation [4, 5]. Minimising the use of CR in paediatric patients therefore appears reasonable. In France, diagnostic reference levels (DRLs) have been determined in the musculoskeletal field for hip radiographs [6].

1. EUR-Lex - 32013L0059 - EN - EUR-Lex. http://eur-lex.europa.eu/legal-content/FR/TXT/?uri=CELEX%3A32013L0059. Accessed 27 Feb 2016

2. Remedios D, Hierath M, Ashford N, et al (2014) Imaging referral guidelines in Europe: now and in the future-EC Referral Guidelines Workshop Proceedings. Insights Imaging 5:9–13. doi: 10.1007/s13244-013-0299-8

3. (2007) ICRP Publication 105. Radiation protection in medicine. Ann ICRP 37:1–63. doi: 10.1016/j.icrp.2008.08.001

4. Ronckers CM, Land CE, Miller JS, et al (2010) Cancer mortality among women frequently exposed to radiographic examinations for spinal disorders. Radiat Res 174:83–90. doi: 10.1667/RR2022.1

5. Wingren G, Hallquist A, Hardell L (1997) Diagnostic X-ray exposure and female papillary thyroid cancer: a pooled analysis of two Swedish studies. Eur J Cancer Prev Off J Eur Cancer Prev Organ ECP 6:550–556.

6. Arrêté du 24 octobre 2011 relatif aux niveaux de référence diagnostiques en radiologie et en médecine nucléaire.
